# Scale Adjustments to Facilitate Two-Dimensional Measurements in OCT Images

**DOI:** 10.1371/journal.pone.0131154

**Published:** 2015-06-25

**Authors:** Marina Garcia Garrido, Regine L. Mühlfriedel, Susanne C. Beck, Christine Wallrapp, Mathias W. Seeliger

**Affiliations:** 1 Division of Ocular Neurodegeneration, Institute for Ophthalmic Research, Centre for Ophthalmology, Tuebingen, Germany; 2 BTG International Germany GmbH, Alzenau, Germany; University of Melbourne, AUSTRALIA

## Abstract

**Purpose:**

To address the problem of unequal scales for the measurement of two-dimensional structures in OCT images, and demonstrate the use of intra¬ocular objects of known dimensions in the murine eye for the equal calibration of axes.

**Methods:**

The first part of this work describes the mathematical foundation of major distortion effects introduced by X-Y scaling differences. Illustrations were generated with CorelGraph X3 software. The second part bases on image data obtained with a HRA2 Spectralis (Heidelberg Engineering) in SV129 wild-type mice. Subretinally and intravitreally implanted microbeads, alginate capsules with a diameter of 154±5 μm containing GFP-marked mesenchymal stem cells (CellBeads), were used as intraocular objects for calibration.

**Results:**

The problems encountered with two-dimensional measurements in cases of unequal scales are demonstrated and an estimation of the resulting errors is provided. Commonly, the Y axis is reliably calibrated using outside standards like histology or manufacturer data. We show here that intraocular objects like dimensionally stable spherical alginate capsules allow for a two-dimensional calibration of the acquired OCT raw images by establishing a relation between X and Y axis data. For our setup, a correction factor of about 3.3 was determined using both epiretinally and subretinally positioned beads (3.350 ± 0.104 and 3.324 ± 0.083, respectively).

**Conclusions:**

In this work, we highlight the distortion-related problems in OCT image analysis induced by unequal X and Y scales. As an exemplary case, we provide data for a two-dimensional *in vivo* OCT image calibration in mice using intraocular alginate capsules. Our results demonstrate the need for a proper two-dimensional calibration of OCT data, and we believe that equal scaling will certainly improve the efficiency of OCT image analysis.

## Introduction

Optical coherence tomography (OCT) has rapidly become an important part of diagnostic imaging in clinical ophthalmology. The continuous advances in spatial resolution have recently also enabled experimental applications in a number of disease models [1, 2]. However, little progress has been made in the development of methods for exact calibration of OCT image data. Indeed, interpretation of the data have been complicated within the last years in part due to the variety of OCT manufacturers, as they all provide their own software [3, 4]. Additionally, differences in their segmentation algorithms on which retinal thickness measurements based on [5, 6], axial resolution in tissue [7], scan density variations [8] and anatomic variations between individual patients as well as inter-species [9, 10] are the most reported obstacles to deal with in the development of methods for exact calibration of OCT image data. In order to study the influence of some of these effects in OCT images, several studies from our lab and other groups worldwide verified retinal layer thickness in OCT scans, measured along the Y axis of images, on the basis of matching histological sections, and established respective correlation coefficients [2, 11, 12]. In OCT data, the Y axis essentially reflects properties of the scan, while the X axis is a product of internal post processing based on a number of inferences. In contrast to those for a histological section, the scales for X and Y in OCT scans are thus not intrinsically identical and need to be calibrated to ensure a system with equal scaling in X and Y (and possibly Z for ‘volume scan’ 3D stacks). Another approach in the attempt to eliminate intra- and intersubject variabilities is the use of a model eye. Agrawal and other groups worked towards the development of an *in vitro* retina phantom for the evaluation of OCT devices [13,14].

In this work, we were looking for a way to obtain equal scales for X and Y *in vivo* that permit two-dimensional measurements in OCT images that are otherwise distorted. As a spherical body has equal dimensions along the X, Y and Z axis, it appears to be a suitable gauge for scaling. Thus, we based our approach on dimensionally stable alginate capsules (Cellbeads) as *in vivo* calibration tool. These beads contain human mesenchymal stem cells transformed to produce green fluorescent protein (GFP) and were placed in the subretinal and intravitreal space of murine eyes as part of the control experiments in a neuroprotection study. The extensive OCT dataset enabled us to estimate the necessary parameters to establish equal X and Y scales, and to provide a mathematical description for the correct measurement of targets contained within an OCT scan in an arbitrary direction. The establishment of equal scales for OCT appears to be essential as it influences both qualitative and quantitative image analysis that often guides diagnosis and treatment in eye diseases [15].

## Materials and Methods

### General remarks

This work is based on data obtained as part of a neuroprotection study. No animal experiments were performed specifically for this project.

### Ethics statement

All procedures were performed according to the German laws governing the use of experimental animals and were previously approved by the local authorities (Regierungspraesidium Tuebingen). In addition, the guidelines set by the Association for Research in Vision and Ophthalmology for the Use of Animals in Ophthalmic and Vision Research were followed during experimentation on animal subjects.

### Animals

The dataset used in this present study included *SV129* wild type mice (n = 12) which were kept under a 12 h: 12 h light-dark cycle (60 lux) and had free access to food and water. They were subcutaneously anesthetized with ketamine (66,7 mg/kg, WDT, Garbsen, Germany) and xylazine (11,7 mg/kg, Bayer, Leverkusen, Germany) and their pupils were dilated with tropicamide eyedrops (Mydriaticum Stulln; Pharma Stulln, Stulln, Germany) prior to microbeads injection and image acquisition.

### Microbeads injections

MicroBeads (CellBeads, CellMed AG, Alzenau, Germany) are alginate microspheres containing human mesenchymal stem cells that may be genetically modified to release therapeutic or marker proteins [16–19]. For this study, mice with MicroBeads that produce eGFP as reporting protein were used [20]. The miniaturized CellBeads for use in mice have a diameter of 154 ± 5 μm, each bead containing about 50–70 GFP-secreting cells. Injections were performed as previously reported [21].

### Spectral domain optical coherence tomography (sd-oct)

Retinal structures of the anesthetized animals were visualized via OCT imaging with a Spectralis HRA+OCT (Heidelberg Engineering GmbH, Heidelberg, Germany). This device features a superluminescent diode at 870 nm as low coherence light source. Scans are acquired at a speed of 40,000 scans per second and each two-dimensional B-scan contains up to 1536 A-scans [2, 11]. The images were taken with the equipment set of 30° field of view and with the software Heidelberg Eye Explorer (HEYEX version 5.3.3.0, Heidelberg, Germany). Resulting images were exported as a 8 bit colour bitmap files and processed with CorelDraw X3 (Corel corporation, Ottawa, ON Canada).

### Scanning-laser ophthalmoscopy (slo)

Eyes were kept moisturized with Methocell (Omnivision, Puchheim, Germany) according to previously described procedures [22]. Briefly, the HRA system features lasers in the short (visible) wavelength range (488 nm and 514 nm), and also in the long (infrared) wavelength range (785/815 nm). The 488 and 795 nm lasers are used for fluorescein (FLA) and indocyanine green (ICG) angiography, respectively. GFP excitation was detected in the autofluorescence mode at 488 nm with a 500 nm barrier filter.

### Statistical analysis

Mean and standard deviation (± SD) values were calculated for the extensions of the microbeads values. The Student's *t*-test was used to analyze statistical significance between epiretinal and subretinal estimated correction factors.

## Results

In a first part, we introduce some theoretical considerations regarding the distortion of retinal structures if, as it appears to be common in OCT setups, the scales for X and Y axes in OCT scans are not identical. In a second part, we show how the scales may be calibrated based on *in vivo* data to avoid such unwanted distortions.

### Part I: Theoretical considerations on the subject of image distortion

**Fig 1:**
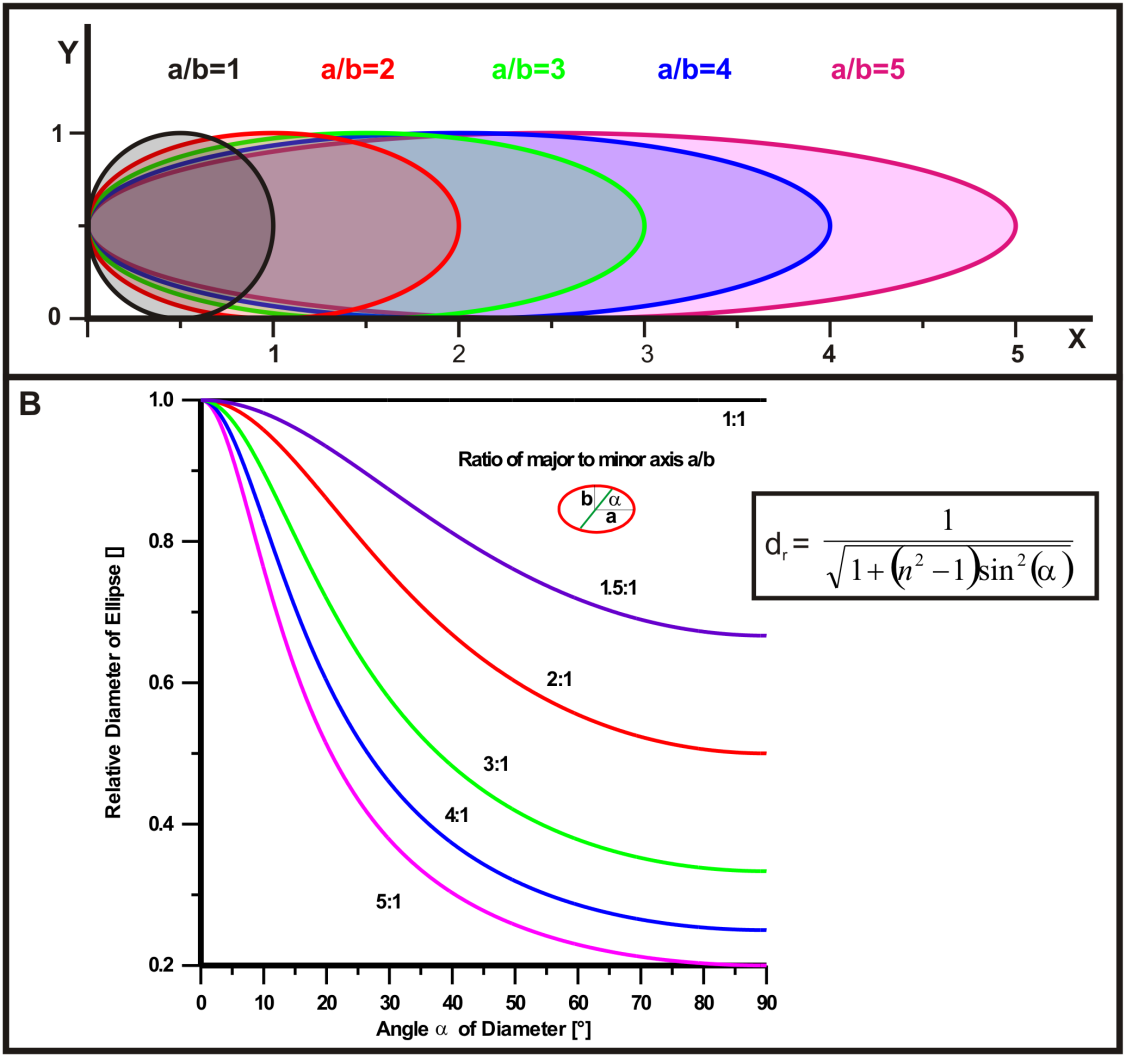
Considerations regarding appearance and cross-section of a circular shape in case of unequal X and Y scales. (A) Illustration of the increasing elliptic appearance of a circle for X/Y ratios above 1. (B) Family of curves representing the relative diameter (d_r_) of an ellipse as a function of the angle of the section (α) for different a/b ratios. A measurement of a cross-section in an unequal X-Y space may be corrected via a division by the respective d_r_.

By design, OCT data consist of many individual scans along the Y axis, while the X axis is a product of internal post processing. Its scaling involves a number of inferences and normative data that may not fit well to the actual application, both in clinical and experimental work. If X and Y axis are not to the same scale, the resulting images will show distortion. We demonstrate this effect here with the help of two examples of defined geometrical shapes. First, we address the behaviour of a circular shape (Fig 1). We assume that the actual object is a circle with the same diameter along X and Y axes (a/b = 1). It is obvious that the circle becomes elliptical when the scale of the X axis changes, here shown up to a radio of a/b = 5 (Fig 1A). Respective retinal structures like e.g. vessels would feature a highly elliptical cross section depending on the magnitude of this effect. When attempting to measure the diameter of the structure, this is only straightforward at 0° or 90°, i.e. entirely along the X or Y axis. While in case of the (original) circle the diameter is independent of the angle of the section, this is not so in case of an ellipse (Fig 1B). Depending on the angle of the section (α) and the ratio between X and Y scales (a/b), the measured result has to be divided by the relative diameter (d_r_) to reflect the correct distance. For a derivation of the formula see Appendix 1. A family of curves for different a/b ratios illustrates this behaviour (Fig 1B).

This effect has to be particularly considered in oblique recordings which are not uncommon in clinical practice. A simple means to avoid any such X-Y distortion and related calculations is to equally calibrate X and Y axes, which we will introduce in a second part below.

**Fig 2:**
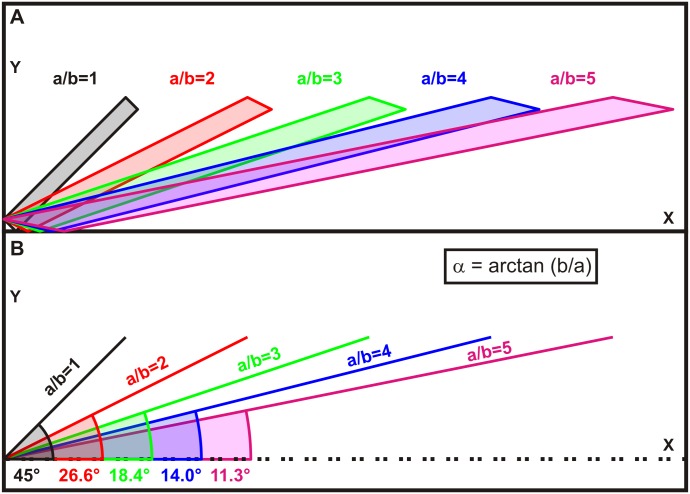
Considerations regarding appearance of a rectangular shape in case of unequal X and Y scales. (A) Illustration of the increasing distortion of a rectangle for X/Y ratios above 1 and the reduction of the off-axis angle. (B) Quantification of the reduction of the off-axis angle for different a/b ratios.

The second example is a rectangular shape at an angle of 45°, which could e.g. reflect a vessel running in the X-Y plane (Fig 2). Again, the scale of the X axis was changed up to ratio a/b = 5 (Fig 2A). It is clear that a change in the scale of X will increase or reduce the angle of the structure; the actual formula for that angle being α = arctan(b/a) (Fig 2B). This effect will lead to an underestimation of the angle when the X scale is larger than the Y scale, and will be a particular problem for any analysis by visual inspection.

### Part II: Use of well-defined intraocular objects for two-dimensional calibration

**Fig 3:**
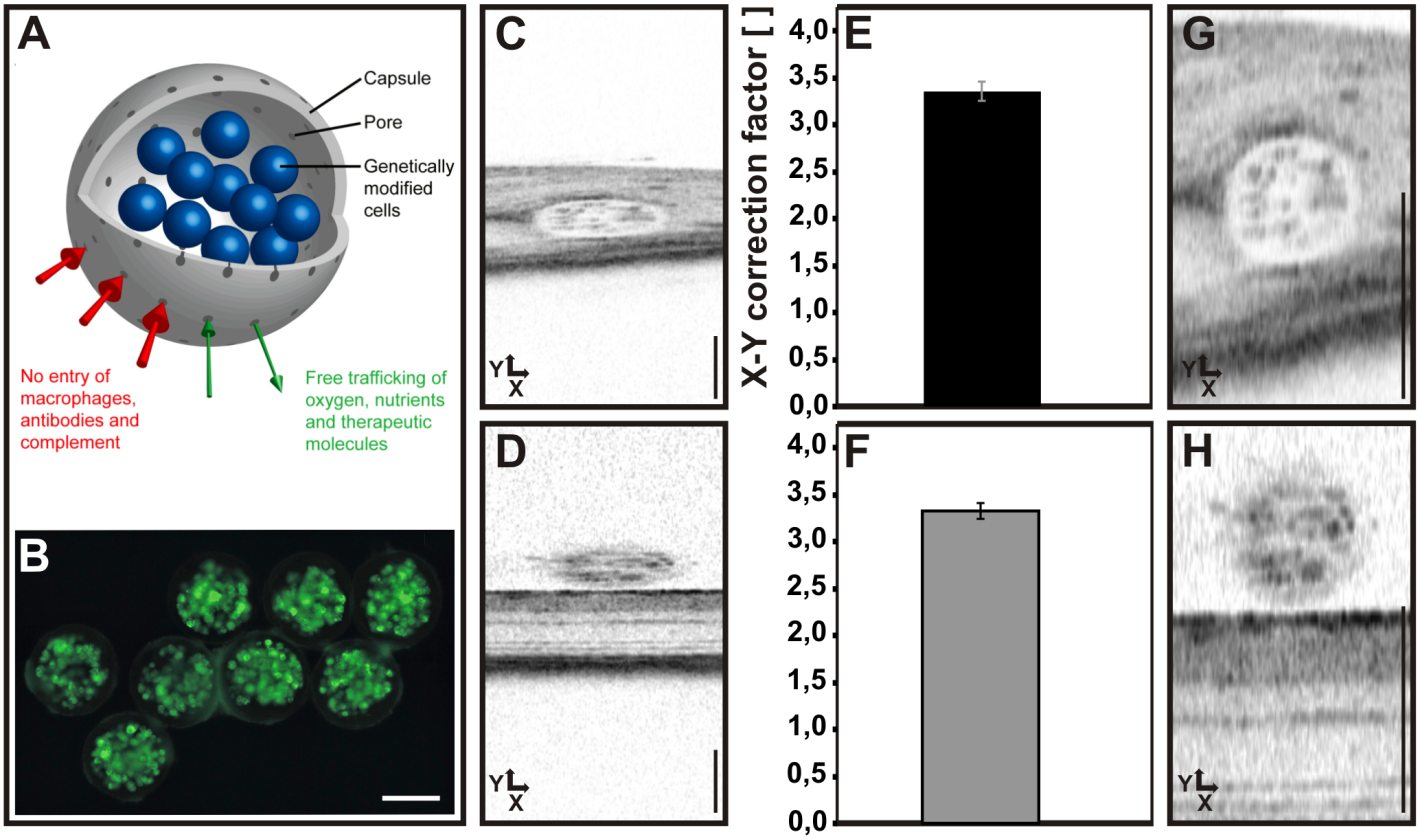
Use of intraocular MicroBeads to determine a correction factor to establish equal X-Y scaling. (A) Schematic drawing of a MicroBead. Encapsulated cells are genetically modified immortalized mesenchymal stem cells which in this case express GFP as a reporter. Scale bar: 100 μm (B). OCT raw images of (C) subretinally and **(D)** epiretinally placed beads. Scale bar: 200 μm. (E, F) Correction factors to establish X-Y equality for subretinal (E) and epiretinal (F) beads. Corrected OCT images of (G) subretinally and (H) epiretinally placed beads. Scale bar: 200 μm. There was no significant difference between E and F (p = 0,57).

As a conclusion from the above results, an equal scaling of X and Y axes appears desirable. Therefore, we assessed the use of well-defined intraocular objects to achieve a two-dimensional calibration of the acquired OCT raw images via an explicit relation between X and Y axis data. We show here that dimensionally stable spherical alginate capsules (CellBeads, Fig 3) are suitable in this regard due to their robust nature and their known size of 154 ± 5 μm with low production tolerances.

**Fig 4:**
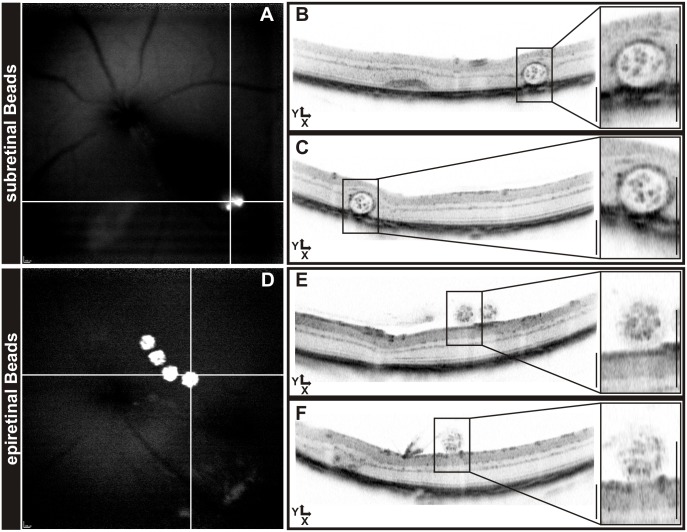
Bead visualization after OCT image correction. Localization of GFP-expressing beads in autofluorescence mode (A, D) and position of horizontal and vertical OCT scans. Using the predetermined correction factor, both subretinal (B, C) and epiretinal beads (E, F) show the correct spherical shape. Scale bars: 200 μm.

The imaging data were obtained as part of a neuroprotection study that included a comparison between subretinal and epiretinal placement of the beads (Figs 3 and 4). Typically, unprocessed raw data (Fig 3C and 3D) show a highly elliptical appearance of both subretinal (Fig 3C) and epiretinal beads (Fig 3D). The original images (.bmp format) acquired with the Heidelberg Eye Explorer software on the Spectralis system were then scaled manually to obtain a spherical shape of the beads (Fig 3G and 3H) with the help of a circular template. We found that, despite a dissimilar appearance of the beads in OCT due to the differences in the environment, there was no perceivable difference in their physical extensions. A correction factor of 3.350 ± 0.104 was found for subretinal beads and of 3.324 ± 0.083 for epiretinally located beads (Fig 3E and 3F), which were not significantly different (p = 0,57). Once the scaling factor has been reliably determined for a certain population, it may be applied to all similar recordings with the respective setup and species. In case a sample to be tested differs greatly from the one in which the correction factor was determined, it would be good to reassure that the factor is valid also for this population. In the specific equipment used here, X and Z scales were found to be largely identical, as can be estimated from X-Z plane sections generated from a 3D ‘volume scan’ dataset. We therefore suggest to scale the Y axis of an OCT scan with the correction factor so that X, Y, and Z have identical scales. An example of images with applied correction (i.e. using the predetermined factor not derived from that specific image) is shown in Fig 4. The known size of the objects further allows to check the absolute calibration and possibly adapt the scales accordingly.

## Discussion

The introduction of the OCT in the clinical practice has without question been one of the major breakthroughs of the recent years. A particular asset of imaging data in general is that a visual inspection is usually sufficient to get an immediate overview of the contained information without the need for an often intransparent numerical analysis. There is a close correlation between OCT data and respective histological sections [2, 10, 23–26] although both are generated quite differently. OCTs are computer-generated images made of a lateral combination of a series of axial reflectivity profiles (A-scans). While the Y axis basically reflects the original A-scans, the X axis is a product of a fitting process based on a number of inferences, so that the scales for X and Y are not intrinsically identical. As scaling properties may be altered by factors like additional lenses in the optical pathway (on the equipment side or on the side of the subject) [27], shape and size of the eye [28], or species differences (in experimental studies) [10, 26], a check of these properties may be required to ensure a system with equal scaling in X and Y (and possibly Z for ‘volume scan’ 3D stacks). It may be for this reason that two-dimensional measurements in OCT images, in contrast to e.g. ultrasound image data, are not widely used so far in clinical practice. The evaluation of OCT data is in the vast majority of clinical applications done by visual inspection, and to a lesser degree also supported by quantitative analysis. Presumably since unequal scales are not part of the natural environment, our visual system is not well suited to incorporate this in the assessment process. We show here that a number of effects may lead to unwanted distortions if the scales for X and Y are not equal, which may influence any conclusions drawn from such images.

**Fig 5:**
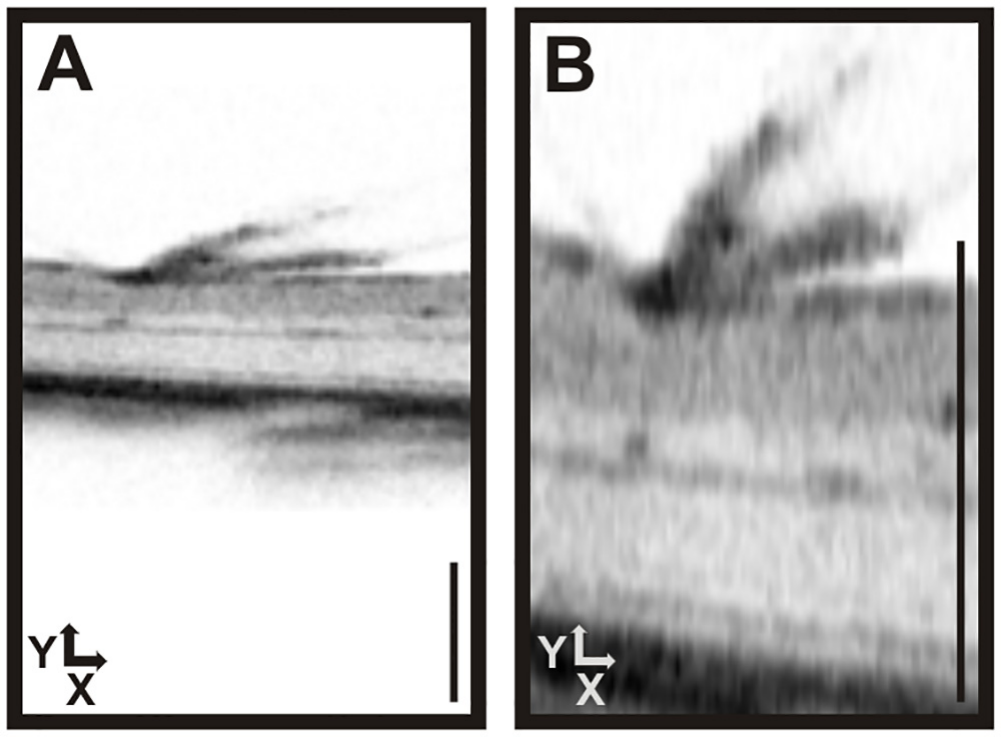
Adjustment of the off-axis angle of a blood vessel running in the X-Y plane. As detailed in Fig 2, the off-axis angle of a retinal vessel is decreased when the X/Y ratio is increased (A). Establishment of equal X-Y calibration, based on the established correction factor, increases the angle to its presumably correct value (B). Scale bars: 200 μm.

The effect of distorted proportions in a two-dimensional graph is demonstrated here on the basis of two examples (Figs 1 and 2). We show that a circular shape turns into an ellipse if X-Y scaling is not equal (Fig 1). A two-dimensional measurement of such a distorted structure would require a correction dependent on the angle of the cross-section as detailed in S1 File. Further, any rectangular structure will appear at an altered angle in the X-Y plane depending on the amount of scale differences (Fig 2), according to the formula given that determines the size of this effect and may be used for a correction (Fig 5).

As a conclusion, we propose to establish an equal scaling of the axes to circumvent the need for any correction of distortion, which would both apply to visual inspection as well as to numerical two-dimensional measurements.

To achieve a two-dimensional calibration of the acquired OCT raw images, a relation between X and Y axis data needs to be established, which in our hands is most reliable when using well-defined intraocular objects *in vivo*. Potentially, this can be anything temporary or persistent from surgical equipment tips, syringes, or fluid droplets to implanted devices like slow release containers or retinal prostheses. In this study, we use spherical MicroBeads containing GFP-expressing mesenchymal stem cells placed either sub- or epiretinally in the retina of *Sv129* wild-type mice. We feel that an *in vivo* verification of scaling is the best option to ensure a proper calibration, as the complexity of the different tissues and pathways are hard to fully integrate in theoretical models or fabricated OCT phantoms [14, 15].

For our setup, a correction factor of about 3.35 was determined from that data (Fig 3). This factor describes the X-Y scaling differences within the same OCT scan and should not be confused with the “conversion constants” between OCT data and histology that have been established in several studies on animal models [2, 11, 12] These studies purely compare the A-Scan data (Y axis) with ex vivo tissue morphometry, whereas the present study is to our best knowledge the first to introduce a method of how a reliable X-Y relationship may be established based on real world data. However, there are limitations in the present study. Although we believe that these findings may be applicable to other OCT devices, the data presented here were acquired with a single OCT system and a device variance was reported already [3, 4]. Additional studies should be needed to confirm this hypothesis.

In summary, we have highlighted the problems in OCT image analysis induced by distortion due to unequal X and Y scales, and we provide an exemplary case for an OCT image calibration based on *in vivo* data using intraocular objects. Our results demonstrate the need for a proper two-dimensional calibration of OCT data, and we expect that the consideration of equal scaling will advance the use of two-dimensional measurements and thereby help to increase the efficiency of OCT image analysis.

## Supporting Information

S1 File Determination of the diameter of an ellipse. (PDF)
