# Supplementary material for: Scale Adjustments to Facilitate Two-Dimensional Measurements in OCT Images
Source: PLoS One. 2015 Jun 25;10(6):e0131154. doi: 10.1371/journal.pone.0131154 (PMC4482384; doi:10.1371/journal.pone.0131154)
Supplement: S1 File — (PDF) [file pone.0131154.s001.pdf]

S1 File. Determination of the diameter of an ellipse

Given: Ellipse with a major axis *a* and a minor axis *b*

Sought: Diameter *d* as a function of the angle α

Approach: If P(x_1,y_1) is the point of intersection between the ellipse and a line through the origin (y=m*x), it follows

$$x_1 = \frac{ab\sqrt{b^2+m^2a^2}}{b^2+m^2a^2}, \text{ and } y_1 = m * x = \frac{mab\sqrt{b^2+m^2a^2}}{b^2+m^2a^2}$$

The radius r (distance between point of origin (0,0) and P) may be calculated as:

$$r = \sqrt{x_1^2 + y_1^2} = \sqrt{\left(1+m^2\right) x_1^2} = \pm x_1\sqrt{\left(1+m^2\right)}$$

Insertion of x_1 renders:

$$r = \sqrt{\left(1+m^2\right) \frac{a^2b^2\left(b^2+m^2a^2\right)}{\left(b^2+m^2a^2\right)^2}} = \sqrt{\left(1+m^2\right) \frac{a^2b^2}{b^2+m^2a^2}}$$

The slope *m* in y=m*x is given as:

$$m = \tan(\alpha) = \frac{\sin(\alpha)}{\cos(\alpha)}$$

Insertion results in:

$$r = \sqrt{\left(1+\frac{\sin^2(\alpha)}{\cos^2(\alpha)}\right) \frac{a^2b^2}{b^2+\frac{\sin^2(\alpha)}{\cos^2(\alpha)}a^2}} = \sqrt{\left(\sin^2(\alpha)+\cos^2(\alpha)\right)\frac{a^2b^2}{a^2\sin^2(\alpha)+b^2\cos^2(\alpha)}}$$

$$= \sqrt{\frac{a^2b^2}{a^2\sin^2(\alpha)+b^2\cos^2(\alpha)}} = \frac{ab}{\sqrt{a^2\sin^2(\alpha)+b^2\cos^2(\alpha)}}$$

Substituting $a = n * b$, it follows:

$$r = \frac{nb^2}{\sqrt{n^2b^2\sin^2(\alpha)+b^2\cos^2(\alpha)}} = \frac{nb}{\sqrt{n^2\sin^2(\alpha)+\cos^2(\alpha)}} = \frac{b}{\sqrt{\sin^2(\alpha)+\frac{1}{n^2}\cos^2(\alpha)}}$$

$\Leftrightarrow$

$$r = \frac{b}{\sqrt{1+\left(\frac{1}{n^2}-1\right)\cos^2(\alpha)}}$$ or alternatively, $$r = \frac{a}{\sqrt{1+\left(n^2-1\right)\sin^2(\alpha)}}$$

The relative diameter d_r of the ellipse equals the radius *r* divided by the long axis *a*. It depends on the ratio n=a/b and is a function of the angle α if $a/b \neq 1$.
